# Polymerase Chain Reaction (PCR)-Negative Herpes Simplex Virus (HSV) Encephalitis in a 62-Year-Old Woman With p-ANCA Vasculitis

**DOI:** 10.7759/cureus.21480

**Published:** 2022-01-21

**Authors:** Ravi Rajmohan, Dina Khoury, Mari Perez-Rosendahl, Lilit Mnatsakanyan, Leonid Groysman

**Affiliations:** 1 Department of Neurology, University of California Irvine, Orange, USA; 2 Department of Psychiatry, University of California Irvine, Orange, USA; 3 Department of Pathology, University of California Irvine, Orange, USA

**Keywords:** case-based review, review, case report, encephalitis, vasculitis, hsv pcr, status epilepticus

## Abstract

We present the case of a 62-year-old woman with a past medical history significant for p-ANCA vasculitis (on immunosuppression) who was found to have polymerase chain reaction (PCR)-negative herpes simplex virus (HSV) encephalitis. We also present a review of all identifiable reports of PCR-negative HSV encephalitis in the past 20 years. To our knowledge, this is the first case of PCR-negative HSV encephalitis in a patient with p-ANCA vasculitis and the thirteenth overall in this timeframe. The patient presented with new-onset fever, encephalopathy, and a first-in-lifetime focal motor seizure progressing to status epilepticus. Cerebrospinal fluid (CSF) PCR was negative for HSV on three separate instances between the first and thirteenth days since symptom onset, and the CSF profile was not typical for HSV encephalitis. The patient underwent a brain biopsy, which confirmed the presence of HSV. She continued to worsen despite aggressive seizure control and six days of empiric acyclovir. Unfortunately, she expired despite the reinitiation of acyclovir. When faced with the classical features of encephalitis in the immunocompromised, the suspicion of HSV should remain high despite negative PCR results. The completion of a full course of acyclovir in the absence of clinical improvement should be considered.

## Introduction

Polymerase chain reaction (PCR) detection of viruses and bacteria has revolutionized diagnostic approaches to infectious diseases. Improvements in technology have made PCR testing a practical and efficient approach to the recognition and management of many life-threatening infections such as herpes simplex virus (HSV) encephalitis [1]. This is in part due to its impressive specificity, cited as being between 95% and 99% with sensitivity between 94% and 98% [2]. However, limitations to these technologies remain and over the past 20 years, several instances of PCR-negative HSV encephalitis have been documented [3-10], raising important questions on how to approach testing such as the timing of testing in relation to symptom onset, need for repeated lumbar punctures, and alternative confirmatory methods. This is especially true in immunocompromised individuals, who are at risk for many variants and obscure entities that may not be detected by standard screening measures. This is of particular concern, as advances in treating autoimmune disorders, organ transplantation, and immunotherapies for cancers have significantly increased the number of patients who are on chronic immunosuppression.

We present the case of a 62-year-old woman with a past medical history significant for systemic lupus erythematosus (SLE) and p-ANCA vasculitis (on immunosuppression) who was found to have PCR-negative HSV encephalitis. We also present a review of all identifiable reports of PCR-negative HSV encephalitis in the past 20 years. To our knowledge, this is the first case of PCR-negative HSV encephalitis in a patient with p-ANCA vasculitis.

## Case presentation

The patient was in her usual state of health when she became febrile to 101°F (38.3 °C).​ The following day, she developed confusion with a leftward head version and leftward gaze deviation. She presented to an outside hospital, where she had multiple episodes of witnessed events concerning for focal motor seizures with progression to generalized bilateral tonic-clonic activity. She was determined to be in status epilepticus and was treated with levetiracetam. Due to concern for meningitis, her initial regimen included methylprednisolone and empiric antibiotic and antiviral coverage with vancomycin, ampicillin, ceftriaxone, acyclovir, and sulfamethoxazole/trimethoprim. Ampicillin was started by the community hospital for empiric coverage of listeria meningitis given the patient’s age and immunocompromised status. Her serum creatinine upon presentation to the community hospital was 1.1 mg/dL, which was slightly worse than her known baseline of 1.04 mg/dl from two weeks prior. She was switched from ampicillin to sulfamethoxazole/trimethoprim to avoid further nephrotoxicity.

**Figure 1:**
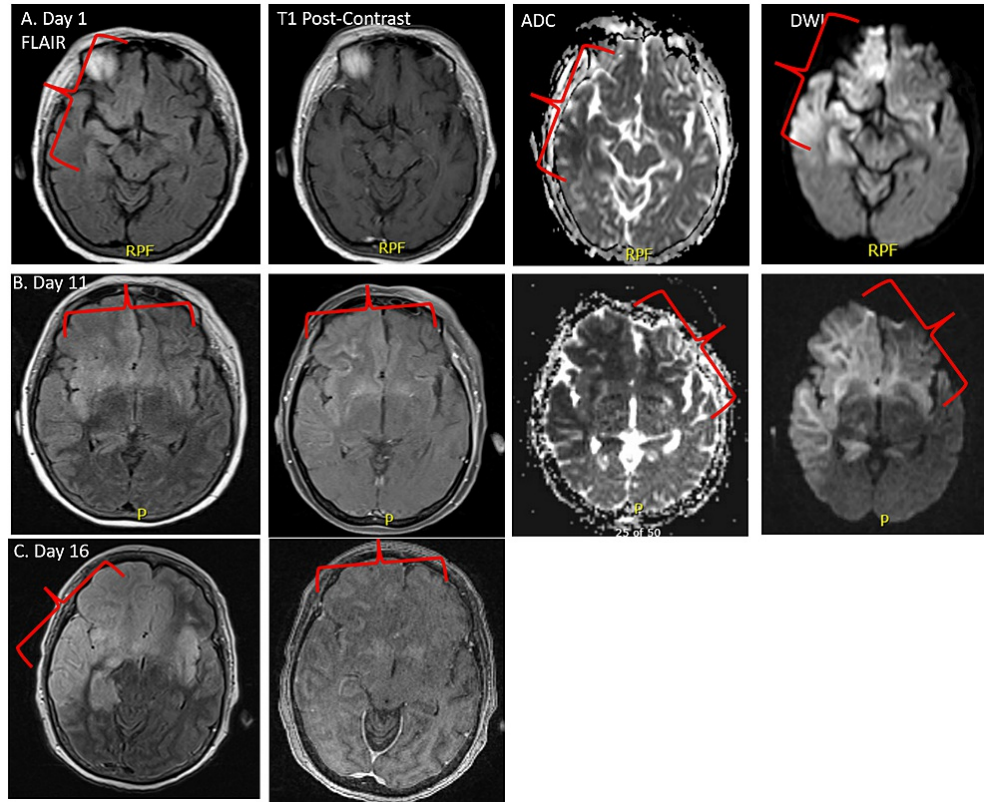
Radiologic progression of disease seen on MRI brain A) Day 1: MRI brain FLAIR demonstrates right temporal and frontal lobe areas of hyperintensity and ADC/DWI mismatch consistent with HSV encephalitis and acute infarction (red arrows). B) Day 11: MRI brain FLAIR shows progression to bilateral involvement. C) Day 16: MRI brain shows severe bilateral frontotemporal enhancement. FLAIR: fluid-attenuated inversion recovery; ADC: apparent diffusion coefficient; DWI: diffusion-weighted imaging; HSV: herpes simplex virus

Routine EEG showed diffuse moderate to severe slowing without epileptiform activity. MRI brain demonstrated nonspecific restricted diffusion in the right basal ganglia and frontal and temporal lobes (Figure 1A). The pertinent laboratory results showed a plasma sodium level of 125 mmol/L and an absolute neutrophil count of 1.1 thous/mcL. Her baseline plasma sodium level was 140 mmol/L and absolute neutrophil count was 5.2 thous/mcL one month prior. Cerebrospinal fluid (CSF) analysis revealed an elevated white blood cell count to 13/mm^3^, a glucose concentration of 78 mg/dL, and a protein level of 41 mg/dL (Table 1). The patient’s blood cultures were negative. Based on available laboratory data, the initial clinical impression was status epilepticus in the setting of hyponatremia. Her neurological status did not improve with the normalization of plasma sodium levels.

**Table 1:** Lumbar puncture results OSH: results obtained from the outside hospital; RBC: red blood cells

Date of Hospital Course	Day 1 (OSH)	Day 5	Day 13
Appearance	Not reported	CLEAR	CLEAR/COLORLESS
RBC CSF (mm^3^)	Not reported	1 (H)	22 (H)​
Nucleated Cells (mm^3^ )	13 (H)	1 ​	1​
CSF Lymphocytes %	Not reported	77	33​
Glucose, CSF ​(mg/dl)	78 (H)	97 (H)	43​
Total Protein, CSF (mg/dl)	41 (H)	49 (H)	62 (H)
Lactate, CSF (mmol/l)	-	3.5 (H)	-
Albumin, CSF (mg/dl)	-	20	-

The patient had a long-standing history of SLE and had been diagnosed with p-ANCA vasculitis two months prior to presentation. She had undergone seven sessions of plasmapheresis since her diagnosis of p-ANCA vasculitis and was maintained on prednisone 60 mg PO daily and cyclophosphamide 50 mg PO daily.

The patient was transferred to a tertiary referral center two days after the start of her symptoms. To address suspected autoimmune and infectious etiologies, an extensive workup was performed (Tables 2-3). Repeat MRI findings on day four were not typical of the changes seen in lupus cerebral vasculitis, leading to further assessment for infectious causes. Another CSF sample obtained five days after symptom onset was not consistent with inflammatory or infectious etiology (Table 1). The patient’s CSF HSV PCR, obtained five days after symptom onset, was negative; thus, her acyclovir was discontinued after six days of treatment.

**Table 2:** Workup for autoimmune etiologies ANA: antinuclear antibody; C3: complement 3 antibody; C4: complement 4 antibody; SSA: Sjogren's syndrome antibody

Test	Days since symptom onset	Result		Test	Days since symptom onset	Result
ANA ab titer	4	80 (+)		Hepatitis C ab	5	Neg.
C3	4	48 (L)		Lupus anticoagulant	5	Weakly positive
C4	4	18		Smith ab, IgG	5	3
Cardiolipin IgG	5	<9		SSA ab, IgG	5	2
Cardiolipin IgM	5	<9.4		SSB ab, IgG	5	2

**Table 3:** Workup for infectious etiologies Cx: culture; ENV: enterovirus; HBV: hepatitis B virus; HIV: human immunodeficiency virus; HSV: herpes simplex virus; WNV: West Nile virus

Test	Days since symptom onset	Result		Test	Days since symptom onset	Result
Bacterial Cx (CSF and serum)	5	Neg.		HSV PCR (CSF)	5	Neg.
Coccidioides Screen (serum)	4	Neg.		Mycobacterial (CSF and serum)	5	Neg.
Cryptococcus Neoformans (serum and CSF)	5	Neg.		Quantiferon mitogen	5	0.12
ENV PCR (CSF)	5	Neg.		Toxoplasma IgG ab	5	3.08 (H)
Fungal Cx (CSF and serum)	5	Neg.		Toxoplasma IgM ab	5	<10
HBV Core a b (serum)	13	Neg.		Treponema Pallidum (serum)	4	Neg.
HBV Surface a b (serum)	13	Pos.		VDRL (CSF)	5	Neg.
Histoplasma	5	Neg.		WNV ab IgG (CSF)	5	0.06
HIV screen (serum)	4	Neg.		WNV ab IgM (CSF)	5	0.02
HSV PCR (Brain Bx)	18	Pos.				

Unfortunately, her condition worsened, and she began to develop multiorgan failure by day six despite aggressive seizure control. A repeat MRI brain on day 11 showed disease progression (Figure 1B) while a third lumbar puncture on day thirteen showed a similar profile to prior results (Table 1).

**Figure 2:**
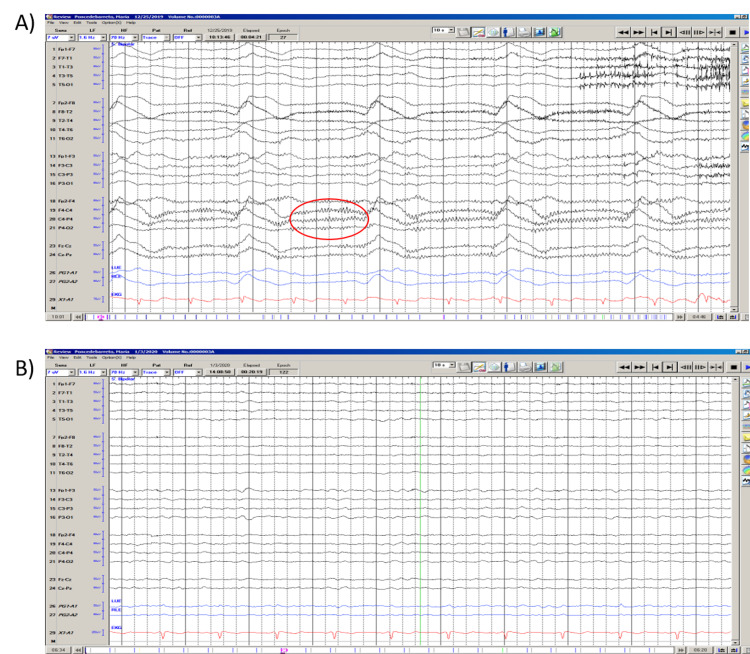
Notable EEG findings A) EEG on presentation to the tertiary center demonstrating right frontal central fast activity (red ellipse), which correlated with left facial twitching on day three of symptom onset; B) Attenuation of EEG by day 13 since symptom onset

Continuous EEG was initiated upon arrival to a tertiary referral center and showed right frontal central area rhythmic fast activity, which correlated with left facial twitching that resolved with treatment with lorazepam and levetiracetam. Afterward, EEG was noted to have diffuse generalized slowing. By day 14, continuous EEG monitoring demonstrated, with sedation held, voltage attenuation with diffuse slowing, and generalized periodic discharges consistent with severe encephalopathy (Figure 2).

**Figure 3:**
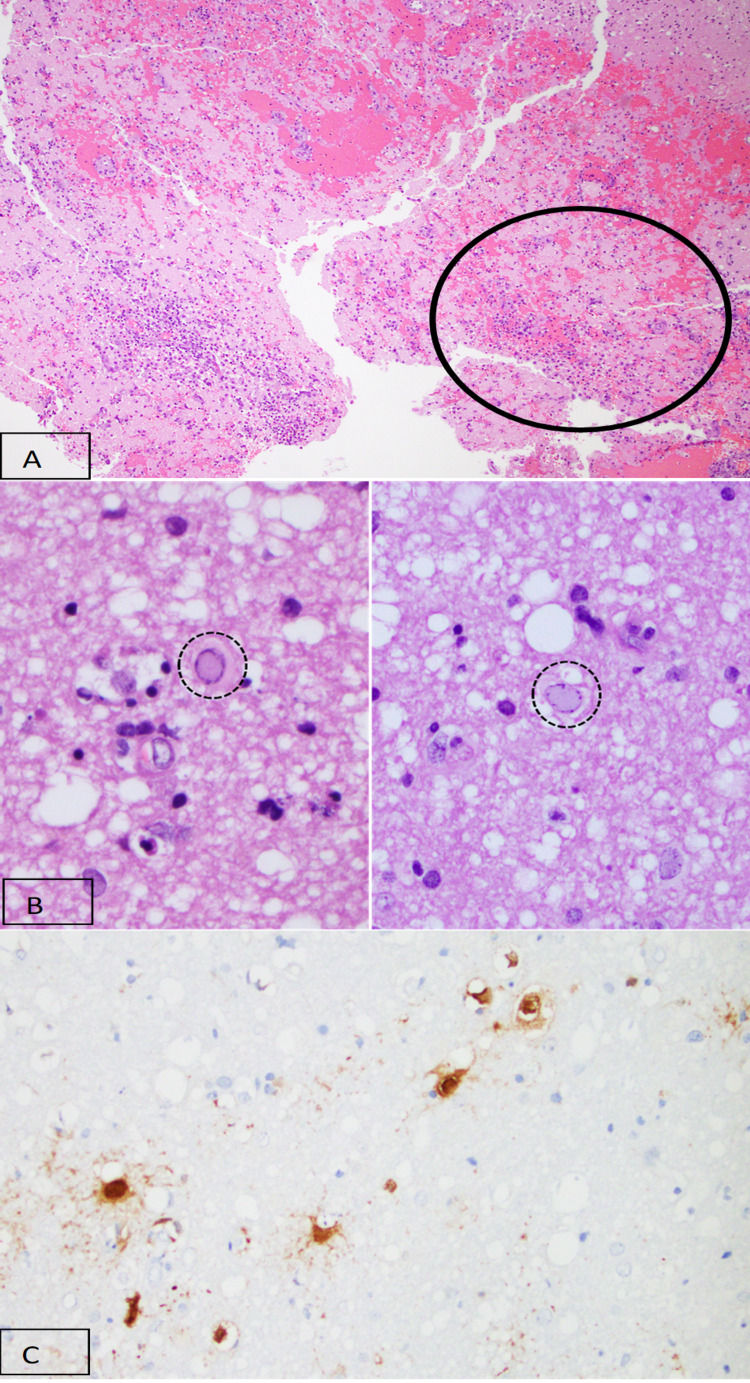
Histopathology of brain biopsy a) H&E stain at 40x magnification shows the extent of hemorrhage (red areas) and non-specific macrophage infiltration (black circle). b) H&E stain at 200x magnification shows an enlargement of the nuclei with margination of chromatin.​ However, this is not specific to viral infection and may be seen in other disorders such as acute hepatic encephalopathy. c) Finally, immunohistochemical staining for HSV at 400x magnification shows the number of cells positive on the HSV1 stain far outnumbered the number of cells that showed viral changes visible on the H&E, which is commonly the case in HSV encephalitis. HSV: herpes simplex virus; H&E: hematoxylin and eosin

A repeat MRI on day 16 showed evolving supra/infra-tentorial infarctions with diffuse sulcal fluid-attenuated inversion recovery (FLAIR) hyperintensities (Figure 1C) prompting brain biopsy (Figure 3). The biopsy showed cortical gray matter with hemorrhagic necrosis, abundant macrophage infiltration, and perivascular/vascular neutrophilic and lymphocytic infiltration of leptomeningeal and cortical grey matter vessels. Herpes simplex virus type 1 was isolated on intraoperative viral cultures and confirmed on immunohistochemical staining of the brain biopsy (Figure 3). Acyclovir was restarted for a 14-day course; however, the patient’s condition continued to deteriorate. On day seven of reinitiating acyclovir, the family elected to change goals of care to comfort only.

## Discussion

Although the specificity of HSV PCR is between 95% and 99% with sensitivity between 94% and 98% [2], our literature review has identified 12 cases of suspected PCR-negative HSV encephalitis published within the last 20 years, thus making this case the thirteenth. Five of these cases were confirmed either via HSV IgG antibodies or repeat HSV PCR [4,7,9]. Only four cases, including the one presented here, have been confirmed with brain biopsy [6,8,10]. A summary of the literature is presented for comparison (Tables 4-5).

**Table 4:** Literature review F: female; FLAIR: fluid-attenuated inversion recovery; G: glucose; L: lymphocytes; LP: lumbar puncture; M: male, N: neutrophils; p: protein; PH: previously healthy; Pt: patient; R: red blood cells; sxs: symptoms; Szs: seizures; W: white blood cells; WNL: within normal limits; y/o: year old

Author, year	Patient characteristics	Presentation	MRI findings	Days after symptom onset CSF was drawn and CSF profile	Method of confirmation of HSV encephalitis	Outcome
Puchhammer-Stockl et al., 2001 [3]	3 patients without the description of demographics	Not described		CSF profiles not reported.	Suspicion based on MRI and clinical evidence of HSV encephalitis	Not reported
	Pt 1 = 82 y/o		Pt 1 = temporal focus	Pt 1=3		
	Pt 2 = 68 y/o		Pt 2 = temporal focus	Pt 2=4		
	Pt 3 = 68 y/o		Pt 3= extended temporal and occipital lobe abnormalities	Pt 3=18		
Denes et al., 2010 [4]	Pt 1 = 54 y/o M	Pt 1 = confusion, Szs, fever to 38.8^0^C	Pt 1 = Signs of encephalitis (day 20)	Pt 1 <2 R 0, W 89 (32% L), P 53, G WNL	CSF antibody testing (anti-HSV immunoglobulins (IgGs))	Not reported
	Pt 2 = 67 y/o F	Pt 2 = confusion, speech disorder, fever to 38^0^C	Pt 2 = Signs of encephalitis (day 3)	Pt 2 <2 R 0, W 610 (96% L), P 124, G WNL		
	Pt 3 = 31 y/o F	Pt 3 = confusion, Szs, fever to 38.5^0^C	Pt 3 = Normal	Pt 3 =10 R 0, W 190 (87% L), P 98, G WNL		
Adler et al., 2011 [5]	35 y/o PHF	HA, Szs, fever to 38.5^0^C	Repeat MRI had increased signal intensity in the medial aspect of the temporal lobes	1^st^ LP = 2 R 3, W 2 (0% L), P 31, G 69	Suspicion based on MRI and clinical evidence of HSV encephalitis	sxs improved with acyclovir; resolved with a 21-day course
				2^nd^ LP = 3 R 17, W 50, P 24, G 76		
Rice et al., 2014 [6]	51 y/o F with distal sigmoid colon cancer treated with resection and chemotherapy 4 years prior	Confusion, aphasia, Szs, fever to 39.4^0^C	Hyperintense FLAIR signal of left temporal lobe	1^st^ LP = 0 (day of sxs onset) R 16, W 20 (85% N), P 44, G 68	Confirmed by brain biopsy	Aphasia persisted, other sxs resolved with a 21-day course
				2^nd^ LP = 6 R 303, W 17 (70% L), P 102, G 60		
Buerger KJ, Zerr K, Salazar R, 2015 [7]	74 y/o M with a history of pulmonary fibrosis and amyloid lung nodules	Acute onset, right-sided hemiparesis, confusion, Szs, fever to 38.5^0^C	Subtle increased T2 signal within the left median frontal lobe seen on contrast brain MRI	1^st^ LP = 1 R 0, W 15 (75% L), P 43, G 60	Positive PCR on 3^rd^ LP	Pt passed away from septic shock
				2^nd^ LP = 9 R 0, W 35 (95% L)		
				3^rd^ LP = 24 R 0, W 17 (32%L), P 48, G 86		
Schuster et al., 2019 [8]	47 y/o M with a history of non-Hodgkin’s lymphoma 5 years prior and B-cell lymphoma 16 years prior (both in remission s/p chemotherapy and radiation)	Behavioral changes, Szs, did not have a fever	Hyperintense FLAIR signal of the left prefrontal gyrus without diffusion restriction	1^st^ LP = 2, W 13, P 574	Confirmed by a brain biopsy	Pt passed due to bowel ischemia from mesenteric artery thrombosis
				2^nd^ LP = 7 W 29, P WNL		
Niksefat et al., 2020 [9]	Pt in 9^th^ decade of life with a history of right corneal transplant secondary to Fuchs' corneal dystrophy.	Behavioral changes, Szs, did not have a fever	T2 signal hyperintensity in the inferior right temporal lobe	1^st^ LP = 1 R 3, W 0, P 55, G 60	Positive PCR on 3^rd^ LP	Pt had near-total resolution except for mild memory problems and imbalance after completion of a 21-day course.
				2^nd^ LP = 4 R18, W3, P 69, G 71		
				3^rd^ LP = 11 R 1, W 20, P 80, G 58		
Roberts et al., 2021 [10]	64 y/o M who consumed 6 to 12 alcoholic drinks per day	Confusion, Szs, fever to 40.8^0^C	T2 hyperintensity in the right medial temporal lobe on MRI	1^st^ LP =1 R 6, W 23 (69% N), P 56, G 55	Positive PCR on 3^rd^ LP Confirmed by brain biopsy	Pt passed away from HSV encephalitis by day 28
				2^nd^ LP = 5 R 0, W 0, P 43, G 44		
				3^rd^ LP =13 R 0, W 15 (50% L), P 187		

**Table 5:** Summary of cases HSV: herpes simplex virus; CSF: cerebrospinal fluid

Demographics	Clinical Presentation (n=10)	Diagnostic findings	Outcomes (n=7)
Age (years) 64 +/- 16	Classic triad of confusion, seizures, and fever (6/10)	MRI (n=14) 8 temporal focus, 5 other abnormalities, 1 normal	Significant improvement with treatment (3/7; 43%)
Males: Females 4:5	Confusion (10/10)	Pleocytic CSF profile 12/18 (66%)	Passed from HSV encephalitis (2/7; 29%)
Pre-existing condition (6/7)	Seizures (9/10)	7/13 (54%) had negative HSV PCR results for samples drawn between 4 and 7 days from symptom onset	Passed from other cause (1/7; 14%)
	Fever >38^0^C (8/10)	Hemorrhagic CSF profile 0/18	

The median age was 64 ± 16 years, with the youngest being 31 and the oldest being 82. The sex of the patient was reported in nine cases with near equal distribution (5 females, 4 males). Only one patient was explicitly stated to have been previously healthy [5], and this case did not have confirmation of HSV by biopsy, IgG, or PCR. Six of the seven patients with reported past medical history, including the one presented here, had some form of potential immunocompromise [6-10]. The classic triad of confusion, fevers, and seizures was seen in six of 10 cases. Overall, clinical signs were consistently observed across cases despite negative PCR results. Confusion was seen in all 10 cases, seizures in nine, and fever >38^0^C in eight. Cortical hyperintensities were noted on 13/14 brain MRIs, demonstrating its role in supporting clinical suspicion. However, five, including this case, noted abnormalities that were not the classic temporal lobe involvement [4-5,7-8].

With the rising recognition of CSF PCR-negative HSV results, the general consensus suggests the virus may not be detectable until day three of illness [3]. It is therefore advised to repeat testing between days three-seven if there is high clinical suspicion in light of a negative result [4]. Eighteen CSF profiles were reported from nine different patients. None showed the typical findings of HSV encephalitis [11] - hemorrhage or xanthochromia [4-10]. However, 12 of the 18 showed elevated white blood cell count in the CSF [4-10]. Furthermore, seven (including this case) of the 13 cases were shown to have a PCR-negative result within the recommended timeframe of three-seven days after symptom onset [3-4,6-8,10].

Our case adds to the growing literature of PCR-negative HSV encephalitis and demonstrates that patients on chronic immunosuppression therapy may have a prolonged PCR-negative period. This is thought to be because these cases may represent the reactivation of latent HSV and not acute infections [8]. Other possibilities include that partial treatment with acyclovir may keep the viral count below the detection threshold for greater than three days [10]. Alternatively, the growing number of reports of PCR-negative HSV encephalitis cases may be due to the rise of mutations occurring within the commercially available primer site; a concern first raised with the earliest reports of PCR-negative HSV encephalitis [3]. Similar to Roberts et al., empiric treatment of acyclovir was held after the second negative PCR result until HSV was confirmed on the biopsy specimen 10 days later [10]. Unfortunately, this may have led to further radiologic and clinical progression of the disease and was ultimately fatal. It is, therefore, crucial to recognize the classical features of HSV encephalitis: fever, altered mental status, and seizures [12] and to consider continuing empiric treatment per IDSA guidelines for a full 14-21-day course [13].

## Conclusions

We present this case and literature review to highlight commonalities in cases of PCR-negative HSV encephalitis. We wish to raise awareness of this uncommon, yet devastating, disease. This presentation is considerably more common in those with pre-existing conditions that may result in immunocompromise. Signs of fever, altered mental status, and seizures are frequently seen among these cases and should raise clinical suspicion. Additionally, cortical hyperintensities are frequently noted on MRI, although the classical presentation of temporal lobe involvement was seen in only half the cases. Cases of PCR-negative HSV encephalitis were commonly seen to have a pleocytic CSF profile but lacked the presence of red blood cells typically seen in CSF. When faced with the classical features of encephalitis, the suspicion of HSV should remain high, even in light of HSV PCR-negative results. The pursuit of brain biopsy and completion of a full course of empiric antiviral treatment should be considered in the absence of clinical improvement or determination of an alternative diagnosis.
